# Transcriptional mechanism of IRF8 and PU.1 governs microglial activation in neurodegenerative condition

**DOI:** 10.1007/s13238-018-0599-3

**Published:** 2018-11-27

**Authors:** Nan Zhou, Kaili Liu, Yue Sun, Ying Cao, Jing Yang

**Affiliations:** 1 State Key Laboratory of Membrane Biology, Peking University, Beijing, 100871 China; 2 IDG/McGovern Institute for Brain Research, Peking University, Beijing, 100871 China; 3 Center for Life Sciences, Peking University, Beijing, 100871 China; 4 School of Life Sciences, Peking University, Beijing, 100871 China; 5 Academy for Advanced Interdisciplinary Studies, Peking University, Beijing, 100871 China; 6 School of Medicine, Tsinghua University, Beijing, 100084 China

**Keywords:** microglia, 3D fluorescence imaging technique, neurodegeneration, IRF8, PU.1

## Abstract

Microglial activation occurs in divergent neuropathological conditions. Such microglial event has the key involvement in the progression of CNS diseases. However, the transcriptional mechanism governing microglial activation remains poorly understood. Here, we investigate the microglial response to traumatic injury-induced neurodegeneration by the 3D fluorescence imaging technique. We show that transcription factors IRF8 and PU.1 are both indispensible for microglial activation, as their specific post-developmental deletion in microglia abolishes the process. Mechanistically, we reveal that IRF8 and PU.1 directly target the gene transcription of each other in a positive feedback to sustain their highly enhanced expression during microglial activation. Moreover, IRF8 and PU.1 dictate the microglial response by cooperatively acting through the composite IRF-ETS motifs that are specifically enriched on microglial activation-related genes. This action of cooperative transcription can be further verified biochemically by the synergetic binding of IRF8 and PU.1 proteins to the composite-motif DNA. Our study has therefore elucidated the central transcriptional mechanism of microglial activation in response to neurodegenerative condition.

**Electronic supplementary material:**

The online version of this article (10.1007/s13238-018-0599-3) contains supplementary material, which is available to authorized users.

## INTRODUCTION

As the specialized immune cells of the central nervous system (CNS), microglia participate in diverse physiological and disease processes. Unlike other glial types, microglia originate from the myeloid-lineage progenitor cells that migrate from the yolk sac into the CNS during the early embryonic stage (Ginhoux et al., 2013; Nayak et al., 2014; Ginhoux and Prinz, 2015; Tay et al., 2016). Microglia exert essential functions in brain development, e.g., in the precise establishment of neural connections (Stephan et al., 2012; Aguzzi et al., 2013; Kettenmann et al., 2013; Schafer and Stevens, 2015; Hong et al., 2016). Also, this glial population is indispensible for maintenance of the neural homeostasis throughout adulthood (Prinz and Priller, 2014; Michell-Robinson et al., 2015; Prinz et al., 2017; Tay et al., 2017). For instance, microglia are involved in the neural control of energy balance, and their malfunction results in obesity and other metabolic disorders (Thaler et al., 2010; Kalin et al., 2015; Valdearcos et al., 2015).

Besides their physiological functions, microglia are activated in response to divergent neuropathological insults (Rivest, 2009; Ransohoff and Cardona, 2010; Finsen and Owens, 2011; Ransohoff and Brown, 2012). Microglial activation has been widely observed in neural injuries, pathogenic infections and neurodegenerative diseases. Activated microglia have long been characterized for their phagocytic clearance of the tissue debris left from neural injuries, which is critical for restricting inflammation and promoting tissue repair (Neumann et al., 2009; Kettenmann et al., 2011; Fu et al., 2014). In addition, microglia are activated to elicit the immune defense for timely containing and eliminating infectious pathogens, e.g., Zika virus and West Nile virus, the failure of which could lead to dreadful consequences (Town et al., 2006; Terry et al., 2012; Lum et al., 2017; Meertens et al., 2017). Furthermore, it has been increasingly appreciated that microglia have the key involvement in neurodegenerative diseases (Perry et al., 2010; Perry and Holmes, 2014; Zuchero and Barres, 2015; Ransohoff, 2016; Colonna and Butovsky, 2017; Wolf et al., 2017). For example, microglia are responsible for the clearance of amyloid peptides, and the deficit of such microglial function could be causative for Alzheimer’s disease (Colonna and Wang, 2016; Ulrich et al., 2017). On the other hand, microglia activated by neurodegenerative cues can release a variety of neurotoxic factors, which collaterally damage neural circuits and thus exaggerate the diseases such as Alzheimer’s disease, Parkinson’s disease, amyotrophic lateral sclerosis and glaucoma (Soto and Howell, 2014; Heppner et al., 2015; Meyer-Luehmann and Prinz, 2015; Ransohoff, 2016; Lall and Baloh, 2017). Our in-depth knowledge of the microglial activation would therefore help reveal therapeutic targets for treatment of the debilitating CNS diseases (Biber et al., 2016; Colonna and Butovsky, 2017; Herz et al., 2017).

Despite extensive studies, one of the central aspects of microglia biology, i.e., the regulation of microglial activation, has been largely uncharacterized. In particular, the transcriptional mechanism governing the transition of resting microglia to their activated state remains to be better understood. In this study, we investigated the microglial response to the traumatic injury-induced neurodegeneration. We exploited the iDISCO (immunolabeling-enabled three-dimensional imaging of solvent-cleared organs) for the 3D fluorescence imaging of microglial activation on the whole-tissue level. We showed that transcription factors IRF8 and PU.1 are both essential for microglial activation, as their specific post-developmental deletion abolishes this process. To explore the underlying transcriptional mechanism, we profiled the genomic landscapes of IRF8 and PU.1 in activated microglia. We revealed that IRF8 and PU.1 directly target the gene transcription of each other, which establishes positive feedback to sustain their highly enhanced expression during microglial activation. Moreover, IRF8 and PU.1 dictate the microglial response by cooperatively acting through the composite IRF-ETS motifs that are specifically enriched on microglial activation-related genes. We further verify such cooperative transcription by demonstrating the synergetic assembly of IRF8 and PU.1 proteins to the composite-motif DNA in the biochemical assays. Altogether, our study has elucidated the central transcriptional mechanism of microglial activation in response to neurodegeneration.

## RESULTS

### 3D fluorescence imaging of microglial activation

**Figure 1:**
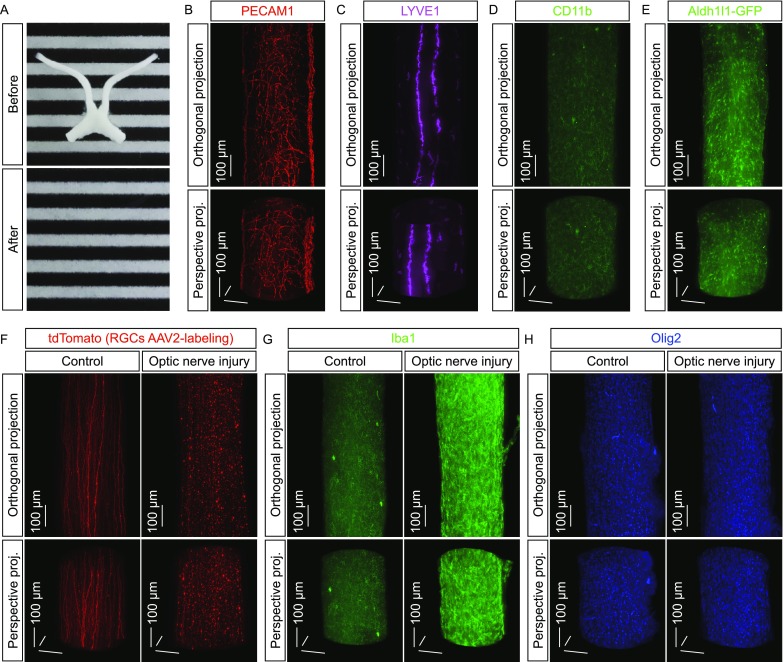
**Three-dimensional fluorescence imaging of microglial activation in response to neurodegeneration**. (A–E) 3D fluorescence imaging of the mouse optic nerves. (A) The optic nerves before (upper panel) and after (lower panel) the iDISCO procedure. (B–D) The optic nerves of wildtype mice were processed for the whole-tissue immunolabeling of PECAM1 (B), LYVE1 (C) or CD11b (D) and imaged on the lightsheet microscope. (E) The optic nerves of *Aldh1l1-GFP* transgenic mice were processed for the whole-tissue immunolabeling of GFP and imaged on the lightsheet microscope. Representative orthogonal (upper panels) or perspective (lower panels) 3D-projection images of the optic nerves are shown. (F) 3D fluorescence imaging of the traumatic injury-induced neurodegeneration. The wildtype mice were intravitreally injected with the tdTomato-expressing AAV2 and then subjected to optic nerve injury. The control (i.e., uninjured) and injured nerves were processed for the whole-tissue immunolabeling of tdTomato and imaged on the lightsheet microscope. Representative orthogonal (upper panels) or perspective (lower panels) 3D-projection images of the optic nerves are shown. (G and H) 3D fluorescence imaging of the glial responses to neurodegeneration. The wildtype mice were subjected to optic nerve injury. The control and injured nerves were processed for the whole-tissue immunolabeling of Iba1 (G) or Olig2 (H) and imaged on the lightsheet microscope. Representative orthogonal (upper panels) or perspective (lower panels) 3D-projection images of the optic nerves are shown

We exploited the model of traumatic injury-induced neurodegeneration to investigate the microglial activation. Axon degeneration after the traumatic nerve injury effectively led to microglial activation in the optic nerves (Yang et al., 2015). To better visualize and accurately quantify this process, we utilized the 3D fluorescence imaging based on the iDISCO technique (Renier et al., 2014). To prove the strength of this imaging approach (Fig. 1A), we firstly visualized the different components of optic nerves. The 3D network of blood vessels, as immunolabeled by the endothelial-cell specific marker PECAM1, was imaged on the whole-tissue level of optic nerves (Fig. 1B). The lymphatic vessels immunolabeled by the lymphatic endothelial marker LYVE1 were also revealed along the pial surface (Fig. 1C and Movie S1). In addition, three major glial types in the optic nerves could be clearly visualized, i.e., microglia by the specific marker CD11b or Iba1 (Fig. 1D and 1G), astrocytes by GFP in *Aldh1l1-GFP* transgenic mice (Fig. 1E), and oligodendrocytes by the specific marker Olig2 (Fig. 1H).

We next examined the microglial response to neurodegeneration. The retinal ganglion cells were sparsely labeled via the intravitreal injection of tdTomato-expressing AAV2. While the tdTomato-labeled optic axons projected through the control (i.e., uninjured) optic nerves, a striking pattern of neurodegeneration occurred at 5 days post-injury (Figs. 1F, S1A and S1B, and Movie S2). Such neurodegenerative condition strongly induced microglial activation as revealed by the whole-tissue immunolabeling of Iba1, i.e., a network of ramified resting microglia was seen in the control optic nerves but the increased density of microglia together with their ameboid appearance became evident at 5 days post-injury (Figs. 1G, S1A and S1C). Of importance, while Iba1 has been the common marker for microglial activation, we found out that the gene expression of *Iba1* (also known as *Aif1*) was directly targeted by IRF8 and PU.1 (See below). Therefore, we chose the alternative marker CD11b to reliably mark out the microglial population through this study. As an aside, the increased density of oligodendrocytes was also observed under the neurodegenerative condition (Figs. 1H and S1D). These results have demonstrated the strength of 3D fluorescence imaging in the whole-tissue assessment of microglial activation.

### IRF8 and PU.1 act in microglial activation under neurodegeneration condition

**Figure 2:**
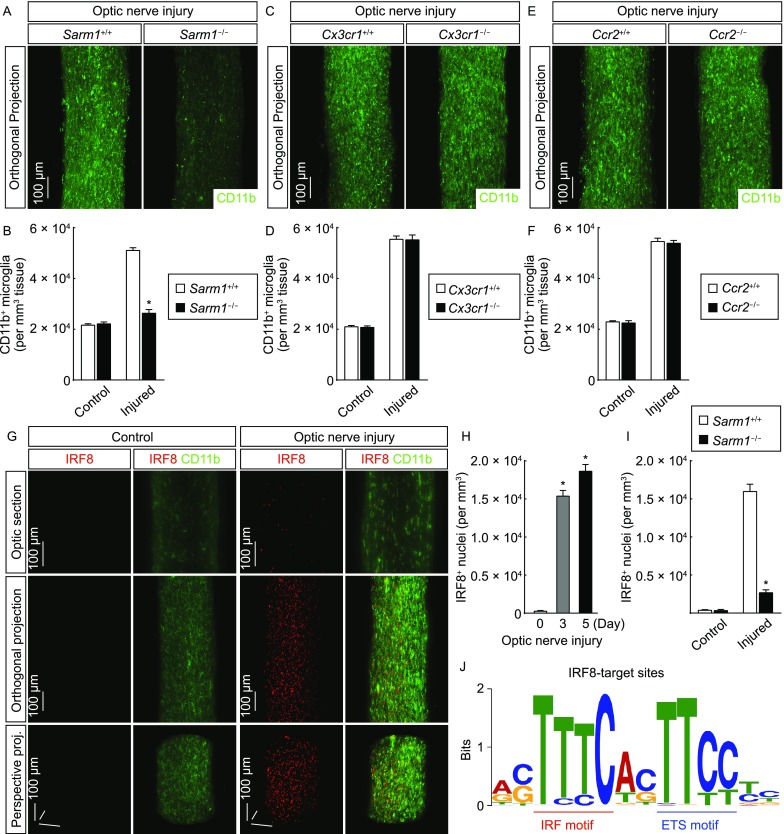
**Transcription factor IRF8 acts in microglial activation**. (A and B) Microglial activation depends on Sarm1-mediated neurodegeneration. *Sarm1*^+/+^ vs. *Sarm1*^−/−^ mice were subjected to optic nerve injury. The control and injured nerves were processed for the whole-tissue immunolabeling of CD11b and imaged on the lightsheet microscope. (A) Representative orthogonal 3D-projection images of the injured nerves. (B) Density of CD11b^+^ microglia. *n* = 4, mean ± SEM, *P < 0.01 (ANOVA test). (C–F) Microglial activation in the injured optic nerves does not depend on the chemokine receptors Cx3cr1 and Ccr2. *Cx3cr1*^+/+^ vs. *Cx3cr1*^−/−^ (C and D) or *Ccr2*^+/+^ vs. *Ccr*^−/−^ (E and F) mice were subjected to optic nerve injury. The control and injured nerves were processed for the whole-tissue immunolabeling of CD11b and imaged on the lightsheet microscope. (C and E) Representative orthogonal 3D-projection images of the injured nerves. (D and F) Density of CD11b^+^ microglia. *n* = 4, mean ± SEM. (G and H) Enhanced expression of transcription factor IRF8 in microglial activation. The wildtype mice were subjected to optic nerve injury. The control and injured nerves were processed for the whole-tissue co-immunolabeling of IRF8 (red)/CD11b (green) and imaged on the lightsheet microscope. (G) Representative optical sections of the lightsheet imaging (upper panels), orthogonal 3D projections (middle panels) or perspective 3D projections (lower panels) of the optic nerves. (H) Density of IRF8^+^ nuclei. *n* = 4, mean ± SEM, *P < 0.01 (Student’s *t*-test). (I) IRF8 up-regulation in microglial activation depends on Sarm1-mediated neurodegeneration. *Sarm1*^+/+^ vs. *Sarm1*^−/−^ mice were subjected to optic nerve injury. The control and injured nerves were processed for the whole-tissue immunolabeling of IRF8 and imaged on the lightsheet microscope. The density of IRF8^+^ nuclei was quantified. *n* = 3, mean ± SEM, *P < 0.01 (ANOVA test). (J) The IRF-ETS composite motif of IRF8 target sites in microglial activation. The wildtype mice were subjected to optic nerve injury, and the injured nerves were processed for ChIP-Seq. The *de novo* analysis of the composite motif of IRF8 target sites was performed. Positions of the IRF motif and ETS motif are highlighted

It was essential to determine whether microglial activation was induced by the neurodegeneration of optic axons or simply by the traumatic injury *per se*. The traumatic injury-induced neurodegeneration, including that in the optic nerves, could be suppressed by genetic deletion of *Sarm1* (Osterloh et al., 2012; Yang et al., 2015). We observed that microglial activation was largely abolished in *Sarm1*^-/-^ mice compared to that in the control mice (Fig. 2A and 2B), suggesting that this microglial response depends on the neurodegenerative condition. We next examined the cellular source involved in microglial activation. Genetic deletion of *Cx3cr1* or *Ccr2*, the two key chemokine receptors for the myeloid-lineage cells, exhibited no detectable effect on microglial activation in *Cx3cr1*^−/−^ vs. *Cx3cr1*^+/+^ mice (Fig. 2C and 2D) or *Ccr2*^−/−^ vs. *Ccr2*^+/+^ mice (Fig. 2E and 2F), indicating that this process likely does not depend on the recruitment of peripheral macrophages or other precursor cells. To further prove this, we exploited the parabiosis procedure. Previous studies documented that the microglial population in the CNS is Cx3cr1^+^ (Goldmann et al., 2013; Parkhurst et al., 2013), which we confirmed in the optic nerves by the complete overlapping of the microglial marker CD11b with the fluorescence EYFP in *Cx3cr1*^*CreERT2-EYFP*/+^ mice (Fig. S4A). We performed the parabiosis procedure between the wildtype and *Cx3cr1*^*CreERT2-EYFP*/+^ mice. The appearance of EYFP-positive cells in the spleen of wildtype mouse in the parabiotic pair showed the success of the procedure (Fig. S1E). However, there were no detectable EYFP-positive cells in the injured optic nerve of the wildtype mouse in the parabiotic pair (Fig. S1F), substantiating that this microglial response does not recruit the circulating population of Cx3cr1^+^ cells. We then determined whether the increased microglial density would be due to local cell proliferation. There was the low expression of Ki67, the specific marker for proliferating cells, in the control optic nerves, but the significant up-regulation of Ki67 occurred in the injured optic nerves (Fig. S2A and S2B). Moreover, the majority of Ki67-immunolabeled nuclei overlapped with the microglial marker (Fig. S2A), demonstrating the local proliferation of microglia in response to neurodegeneration.

We sought to understand the transcriptional mechanism underlying microglial activation. Prior studies reported that transcription factor IRF8 is involved in microglial activation (Horiuchi et al., 2012; Masuda et al., 2012; Minten et al., 2012). We observed that IRF8 expression was almost undetectable in the control optic nerves by the 3D fluorescence imaging (Fig. 2G and 2H). In contrast, the highly enhanced expression of IRF8 appeared as early as at 3 days post-injury (Fig. 2G and 2H). Notably, the IRF8-immunolabeled nuclei exclusively overlapped with the microglial marker (Fig. 2G). In addition, IRF8 up-regulation was strongly inhibited in the injured optic nerves of *Sarm1*^−/−^ mice (Fig. 2I), showing that this IRF8 response depends on the neurodegenerative condition. We then set out to profile the genomic landscape of IRF8 in the activated microglia. We optimized the procedure of chromatin immunoprecipitation (ChIP) for nerve tissues without the necessity of cell isolation, taking advantage of the microglia-specific IRF8 expression. The ChIP-Seq analysis successfully identified 851 IRF8 target sites across the genome (Fig. S2C and S2D). Interestingly, through the *de novo* motif analysis of these IRF8 target sites, we observed not only the consensus interferon regulatory factor (IRF)-binding motif TTTC but also a different, highly represented motif TTCC at two base pairs downstream of the IRF motif (Fig. 2J). In fact, this composite motif (TTCCNNTTTC) was present in 80% of IRF8 target sites (678 out of 851), implying the cooperative action of IRF8 with an additional transcription factor.

**Figure 3:**
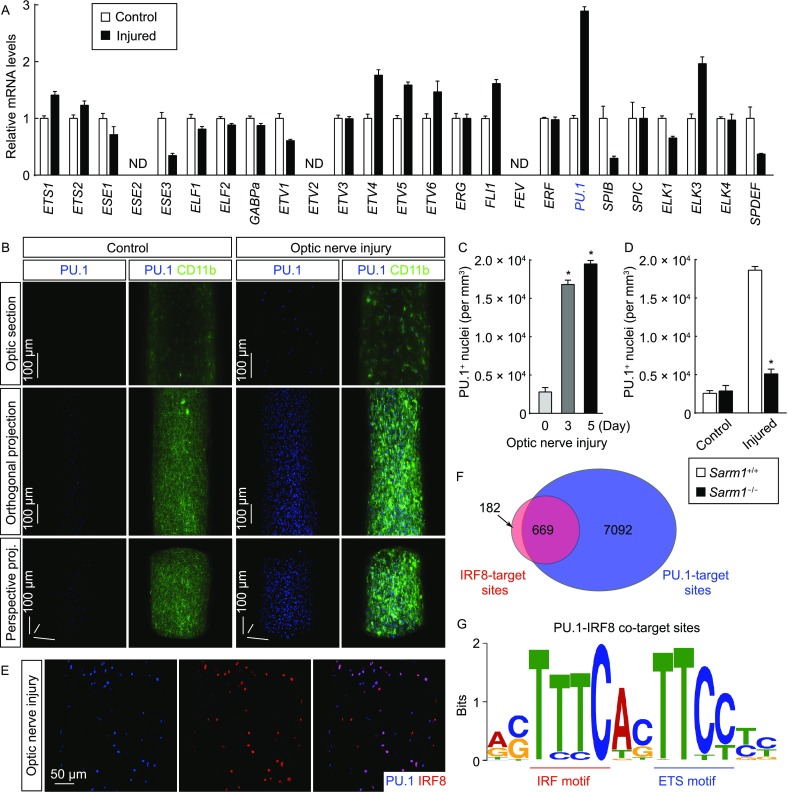
**Transcription factor PU.1 participates in microglial activation**. (A) Expression of ETS-family transcription factors in the optic nerves. The wildtype mice were subjected to optic nerve injury. The control and injured nerves were processed for the qPCR analysis of ETS-family transcription factors. ND, not detected; *n* = 4, mean ± SEM. (B and C) Enhanced expression of PU.1 in microglial activation. The wildtype mice were subjected to optic nerve injury. The control and injured nerves were processed for the whole-tissue co-immunolabeling of PU.1 (blue)/CD11b (green) and imaged on the lightsheet microscope. (B) Representative optical sections of the lightsheet imaging (upper panels), orthogonal 3D projections (middle panels) or perspective 3D projections (lower panels) of the optic nerves. (C) Density of PU.1^+^ nuclei. *n* = 4, mean ± SEM, *P < 0.01 (Student’s *t*-test). (D) PU.1 up-regulation in microglial activation depends on Sarm1-mediated neurodegeneration. *Sarm1*^+/+^ vs. *Sarm1*^−/−^ mice were subjected to optic nerve injury. The control and injured nerves were processed for the whole-tissue immunolabeling of PU.1 and imaged on the lightsheet microscope. The density of PU.1^+^ nuclei was quantified. *n* = 3, mean ± SEM, *P < 0.01 (ANOVA test). (E) Co-expression of IRF8 and PU.1 in microglial activation. The wildtype mice were subjected to optic nerve injury, and the injured nerves were examined by immunohistochemistry for IRF8 and PU.1. (F and G) Cooperative transcription of IRF8 and PU.1 in microglial activation. The wildtype mice were subjected to optic nerve injury, and the injured nerves were processed for the ChIP-Seq analysis of PU.1 target sites. (F) Overlap of PU.1 target sites with the IRF8 target sites. (G) The composite motif of PU.1 target sites overlapping with IRF8 target sites was analyzed. Positions of the IRF motif and ETS motif are highlighted

Bioinformatics suggested this composite motif as the potential binding site of the ETS (E26 transformation-specific) family of transcription factors (Sharrocks, 2001; Hollenhorst et al., 2011). We therefore determined the expression profile of these ETS members in the optic nerves. The mRNAs of the majority of ETS members were detectable, and importantly, *Pu.1* (also known as *Spi1*) exhibited the most significant up-regulation after the nerve injury (Fig. 3A). In accordance with this observation, PU.1 expression was low in the control optic nerves but became highly enhanced as early as at 3 days post-injury, as visualized by the 3D fluorescence imaging (Fig. 3B and 3C, and Movie S3). Similar to the microglia-specific expression of IRF8, the PU.1-immunolabeled nuclei also completely overlapped with the microglial marker (Fig. 3B and Movie S3). In addition, PU.1 up-regulation depended on the neurodegenerative condition, as it was largely abolished in *Sarm1*^−/−^ mice (Fig. 3D).

In light of these results, we hypothesized that PU.1 might be the putative transcription factor cooperating with IRF8 in microglial activation. To support this possibility, we observed that >90% of the PU.1-immunolabeled nuclei were positive for IRF8 expression, and *vice versa*, in the injured optic nerves (Fig. 3E). We then determined the genomic landscape of PU.1 in the activated microglia by the ChIP-Seq procedure, again taking advantage of the microglia-specific PU.1 expression. The ChIP-Seq analysis identified 7,761 PU.1 target sites across the genome (Fig. S3A and S3B). Strikingly, there was a significant overlap of IRF8 target sites (669 out of 851) with PU.1 target sites (Figs. 3F and S3C). Furthermore, the *de novo* motif analysis of these PU.1 target sites overlapping with IRF8 target sites resulted in the composite IRF-ETS motif highly resembling that identified from the IRF8 target sites alone (Fig. 3G), suggesting the cooperative transcriptional action of IRF8 and PU.1 in the microglial response to neurodegeneration.

### Cross-regulation of IRF8 and PU.1 in microglial activation

**Figure 4:**
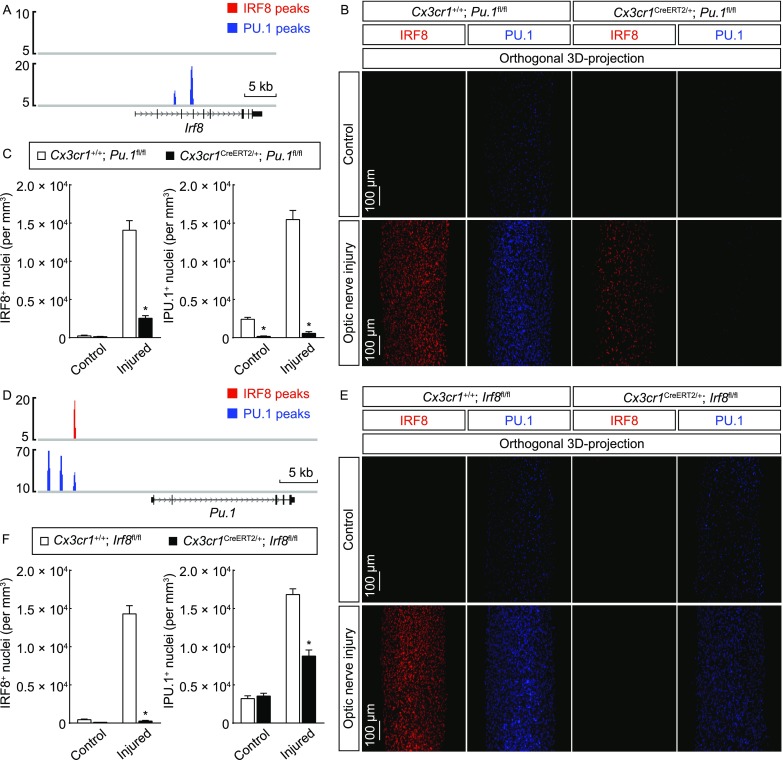
**Cross-regulation of IRF8 and PU.1 in microglial activation**. (A) PU.1 directly targets IRF8 expression in microglial activation. IRF8 and PU.1 target sites at the gene locus of *Irf8* are shown. (B and C) PU.1 regulates IRF8 expression. *Cx3cr1*^+/+^; *Pu.1*^*fl*/*fl*^ vs. *Cx3cr1*^*CreERT2*/+^; *Pu.1*^*fl*/*fl*^ mice were treated with 4-hydroxytamoxifen to induce the Cre-recombinase activity and then subjected to optic nerve injury. The control and injured nerves were processed for the whole-tissue immunolabeling of IRF8 or PU.1 and imaged on the lightsheet microscope. (B) Representative orthogonal 3D-projection images of the optic nerves. (C) Density of IRF8^+^ or PU.1^+^ nuclei. *n* = 3, mean ± SEM, *P < 0.01 (ANOVA test). (D) IRF8 directly targets PU.1 expression in microglial activation. IRF8 and PU.1 target sites at the UREs of *Pu.1* gene locus are shown. (E and F) IRF8 feedback regulates PU.1 expression. *Cx3cr1*^+/+^; *Irf8*^*fl*/*fl*^ vs. *Cx3cr1*^*CreERT2*/+^; *Irf8*^*fl*/*fl*^ mice were treated with 4-hydroxytamoxifen to induce the Cre-recombinase activity and then subjected to optic nerve injury. The control and injured nerves were processed for the whole-tissue immunolabeling of IRF8 or PU.1 and imaged on the lightsheet microscope. (E) Representative orthogonal 3D-projection images of the optic nerves. (F) Density of IRF8^+^ or PU.1^+^ nuclei. *n* = 3, mean ± SEM, *P < 0.01 (ANOVA test)

To decipher the transcriptional mechanism of IRF8 and PU.1, we noticed that there were two PU.1 target sites at the gene locus of *Irf8*, while there was no detectable IRF8 target site at this locus (Fig. 4A). This observation suggested that PU.1 could directly target IRF8 expression in microglial activation. To test this possibility, we bred *Cx3cr1*^*CreERT2-EYFP*/+^; *Pu.1*^*fl*/*fl*^ mice to achieve the microglia-specific deletion of PU.1 in adult mice. The efficiency of the Cre-recombinase activity induced by 4-hydroxytamoxifen was confirmed by the complete overlap of the microglial marker with the Cre-dependent fluorescence tdTomato in *Cx3cr1*^*CreERT2-EYFP*/+^; *Rosa26-LSL-tdTomato* mice (Fig. S4B). Of note, we observed the significant decrease of microglial population in the optic nerves, as well as in other CNS regions, about two weeks after the Cre-induction in *Cx3cr1*^*CreERT2*/+^; *Pu.1*^*fl*/*fl*^ mice (data not shown). This phenomenon could reflect the microglial death due to the gradual depletion of PU.1 protein after the gene knockout, given the essential function of PU.1 in the myeloid-lineage cells (Scott et al., 1994; McKercher et al., 1996; Kierdorf et al., 2013). To circumvent this issue, the mice were subjected to the optic nerve injury immediately after the daily treatment of 4-hydroxytamoxifen for 6 days. There was robust PU.1 up-regulation in the injured optic nerves of control *Cx3cr1*^+/+^; *Pu.1*^*fl*/*fl*^ mice, but this PU.1 response was abolished in *Cx3cr1*^*CreERT2-EYFP*/+^; *Pu.1*^*fl*/*fl*^ mice, confirming the effectiveness of genetic deletion (Fig. 4B and Fig. 4C). More importantly, IRF8 up-regulation was significantly blunted in *Cx3cr1*^*CreERT2-EYFP*/+^; *Pu.1*^*fl*/*fl*^ mice compared to that in control *Cx3cr1*^+/+^; *Pu.1*^*fl*/*fl*^ mice (Fig. 4B and 4C), demonstrating that PU.1 regulates IRF8 expression in this microglial response.

Several UREs (upstream regulatory elements) exert control over the *Pu.1* gene locus. These UREs can be directly bound by PU.1, which functions as the auto-regulatory mechanism of this transcription factor (Li et al., 2001; Okuno et al., 2005). Indeed, three distinct PU.1 target sites were observed at −14 kb, −12 kb and −10 kb of the *Pu.1* locus, proving the quality of our ChIP-Seq analysis (Fig. 4D). Interestingly, we also noticed the presence of an IRF8 target site at −10 kb of the *Pu.1* locus, raising the possibility that IRF8 might feedback regulate PU.1 expression in microglial activation. We therefore bred *Cx3cr1*^*CreERT2-EYFP*/+^; *Irf8*^*fl*/*fl*^ mice to achieve the microglia-specific deletion of IRF8 in adult mice, circumventing the developmental defect of microglia in *Irf8*^−/−^ mice (Kierdorf and Prinz, 2013; Kierdorf et al., 2013). As expected, while there was significant IRF8 up-regulation in the injured optic nerves of control *Cx3cr1*^+/+^; *Irf8*^*fl*/*fl*^ mice, this IRF8 response was completely abolished in *Cx3cr1*^*CreERT2-EYFP*/+^; *Irf8*^*fl*/*fl*^ mice, showing the efficient deletion of IRF8 expression (Fig. 4E and 4F). Moreover, the enhanced expression of PU.1 was largely mitigated in *Cx3cr1*^*CreERT2-EYFP*/+^; *Irf8*^*fl*/*fl*^ mice (Fig. 4E and 4F). These results have together uncovered the cross-regulation of IRF8 and PU.1 in microglial activation under the neurodegenerative condition.

### IRF8 and PU.1 cooperatively dictate microglial activation

**Figure 5:**
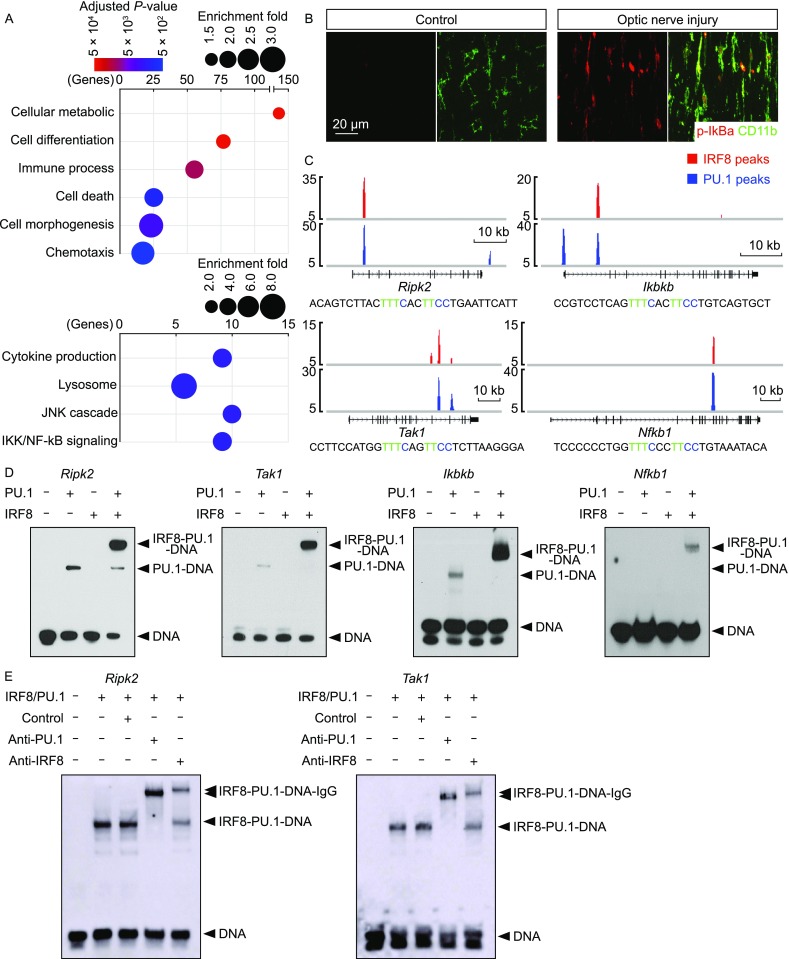
**IRF8 and PU.1 cooperatively target microglial activation-related genes**. (A) IRF8 and PU.1 cooperatively regulate microglial activation-related genes. The genes that contained the IRF8-PU.1 co-target sites were subjected to GO enrichment analysis. Enriched biological processes and signaling pathways are shown. (B) Activation of the NF-κB pathway in microglial activation. Wild-type mice were subjected to optic nerve injury. The control and injured nerves were examined by immunohistochemistry for p-IκBα and CD11b. (C) IRF8 and PU.1 directly target the key signaling components of the NF-κb pathway. IRF8 and PU.1 target sites at the indicated gene loci are shown. The sequence of the DNA probe derived from the IRF8-PU.1 co-target site at each locus is included, with the IRF-ETS composite motif highlighted. (D and E) Biochemical assembly of the ternary complex of the composite-motif DNA with IRF8 and PU.1 proteins. (D) Synergetic binding of IRF8 and PU.1 to the composite-motif DNA, derived from the gene loci of *Ripk2*, *Tak1*, *Ikbkb* or *Nfkb1*, was examined by EMSA. (E) Supershift of the composite-motif DNA/IRF8/PU.1 ternary complex with anti-IRF8 or anti-PU.1 antibody

To further understand the transcriptional mechanism of microglial activation, IRF8 and PU.1 target sites in the genome were analyzed in detail. A total of 331 genes were identified to contain the IRF8-PU.1 co-target sites in the coding and/or regulatory regions (Table S1). The Gene Ontology (GO) enrichment analysis of these co-targeted genes revealed significant enrichment in several biological processes critical for microglial activation (Colonna and Butovsky, 2017; Li and Barres, 2018), e.g., cellular metabolism, cell differentiation, immune process, cell morphogenesis, chemotaxis and phagocytosis (Fig. 5A). In addition, the JNK and NF-κB signaling pathways, which are known for their essential roles in immune response (Vallabhapurapu and Karin, 2009; Arthur and Ley, 2013; Zhang et al., 2017), were also enriched (Fig. 5A).

To verify the cooperative transcriptional action of IRF8 and PU.1, we chose the NF-κB pathway as an example. The NF-κB pathway was strongly activated in the microglial response to neurodegeneration, as assessed by the increased p-IκBα immunostaining (Figs. 5B and S4C). Several key signaling components of the NF-κB pathway, i.e., *Ripk2*, *Tak1*, *Ikbkb* and *Nfkb1*, were identified to harbor the IRF8-PU.1 co-target sites containing the IRF-ETS composite motifs (Fig. 5C). We performed the electrophoretic mobility shift assay (EMSA) to biochemically examine the assembly of a ternary complex of recombinant IRF8 and PU.1 proteins with the composite-motif DNA. IRF8 or PU.1 alone exhibited weak or undetectable interaction with the DNA probes of *Ripk2*, *Tak1*, *Ikbkb* or *Nfkb1* (Fig. 5D). Importantly, simultaneous inclusion of IRF8 and PU.1 proteins in the *in vitro* reactions led to the synergetic binding of IRF8 and PU.1 with each probe tested (Fig. 5D), showing the cooperative action of the two transcription factors in targeting the IRF-ETS composite motif. To further prove the specificity of this ternary-complex formation, we performed the supershift assay of the composite motif/IRF8/PU.1 complex with anti-IRF8 or anti-PU.1 antibody. Addition of the control IgG exhibited no effect on the ternary complex with *Ripk2* or *Tak1* probes (Fig. 5E). In contrast, addition of anti-IRF8 or anti-PU.1 IgG both resulted in the supershift of the composite motif/IRF8/PU.1 ternary complex to the higher molecular weight (Fig. 5E), confirming the presence of IRF8 and PU.1 proteins in the biochemically-assembled complex. These results have demonstrated the cooperative action of IRF8 and PU.1 in targeting the composite IRF-ETS motif in microglial activation.

**Figure 6:**
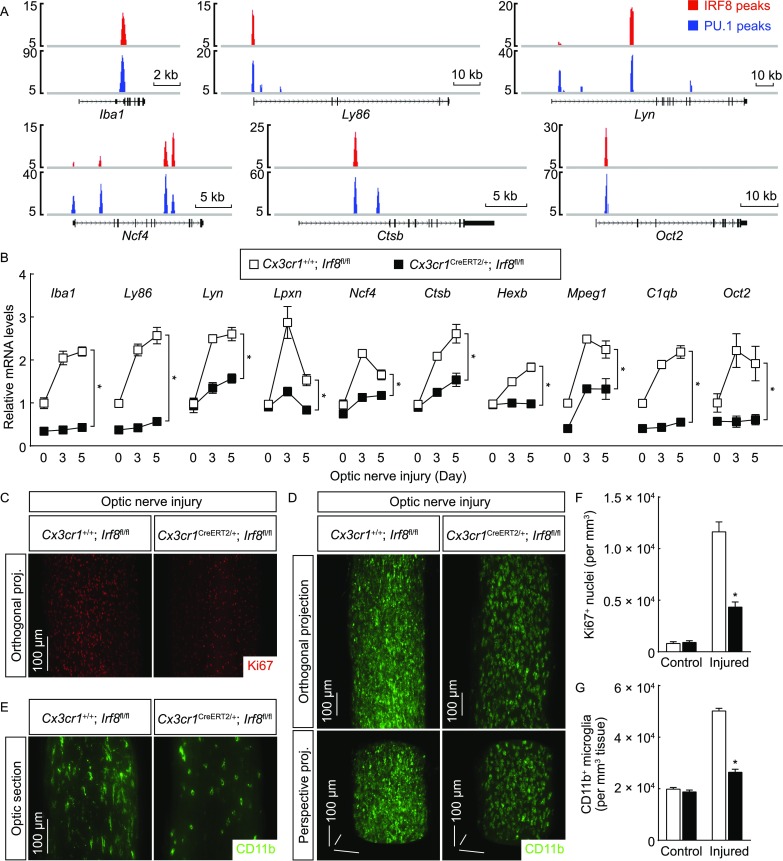
**Post-developmental IRF8 is required for microglial activation**. (A) IRF8 and PU.1 cooperatively target microglial activation-related genes. IRF8 and PU.1 target sites at the indicated gene loci are shown. (B–G) Post-developmental IRF8 is required for microglial activation. *Cx3cr1*^+/+^; *Irf8*^*fl*/*fl*^ vs. *Cx3cr1*^*CreERT2*/+^; *Irf8*^*fl*/*fl*^ mice were treated with 4-hydroxytamoxifen to induce the Cre-recombinase activity and then subjected to optic nerve injury. (B) Expression levels of the indicated genes in the optic nerves were examined by qPCR analysis. *n* = 4, mean ± SEM, *P < 0.01 (ANOVA test). (C to G) The control and injured nerves were processed for the whole-tissue immunolabeling of Ki67 (C and F) or CD11b (D–G) and imaged on the lightsheet microscope. (C) Representative orthogonal 3D projections of the Ki67-immunolabeled injured nerves. (F) Density of Ki67^+^ nuclei. *n* = 3, mean ± SEM, *P < 0.01 (ANOVA test). (D) Representative orthogonal (upper panels) or perspective (lower panels) 3D projections of the CD11b-immunolabeled injured nerves. (E) Representative optical sections of the CD11b-immunolabeled injured nerves. (G) Density of CD11b^+^ microglia. *n* = 4, mean ± SEM, *P < 0.01 (ANOVA test)

To determine the central role of this transcriptional mechanism of IRF8 and PU.1 in microglial activation, a collection of microglial activation-related genes were chosen, e.g., the common marker for microglial activation (*Iba1*), immune process (*Ly86*, *Mpeg1* and *C1qb*), cell morphogenesis (*Lyn* and *Lpxn*), phagocytosis (*Ncf4*, *Ctsb* and *Hexb*) and cell differentiation (*Oct2*). We showed that all of these genes contained the IRF8-PU.1 co-target sites with the composite IRF-ETS motifs (Figs. 6A and S5A). Expression levels of the genes were significantly up-regulated in the injured optic nerves of *Cx3cr1*^+/+^; *Irf8*^*fl*/*fl*^ mice, but such response was suppressed in *Cx3cr1*^*CreERT2-EYFP*/+^; *Irf8*^*fl*/*fl*^ mice (Fig. 6B). In line with this suppression of microglial activation-related genes, the microglial proliferation was also inhibited in *Cx3cr1*^*CreERT2-EYFP*/+^; *Irf8*^*fl*/*fl*^ mice compared to *Cx3cr1*^+/+^; *Irf8*^*fl*/*fl*^ mice, as assessed by the 3D fluorescence imaging of Ki67^+^ nuclei (Fig. 6C and 6F). Accordingly, while the density of microglial population in the control optic nerves was indistinguishable between *Cx3cr1*^+/+^; *Irf8*^*fl*/*fl*^ vs. *Cx3cr1*^*CreERT2-EYFP*/+^; *Irf8*^*fl*/*fl*^ mice, the increase of microglial population was largely blunted in the injured optic nerves of *Cx3cr1*^*CreERT2-EYFP*/+^; *Irf8*^*fl*/*fl*^ mice (Fig. 6D and 6G). In addition, the IRF8-deficient microglia exhibited a stumpy morphology with fewer cellular processes in the injured optic nerves (Fig. 6E), likely reflecting the fact that IRF8 directly targets the pathway of cell morphogenesis as identified by the GO analysis (Fig. 5A).

**Figure 7:**
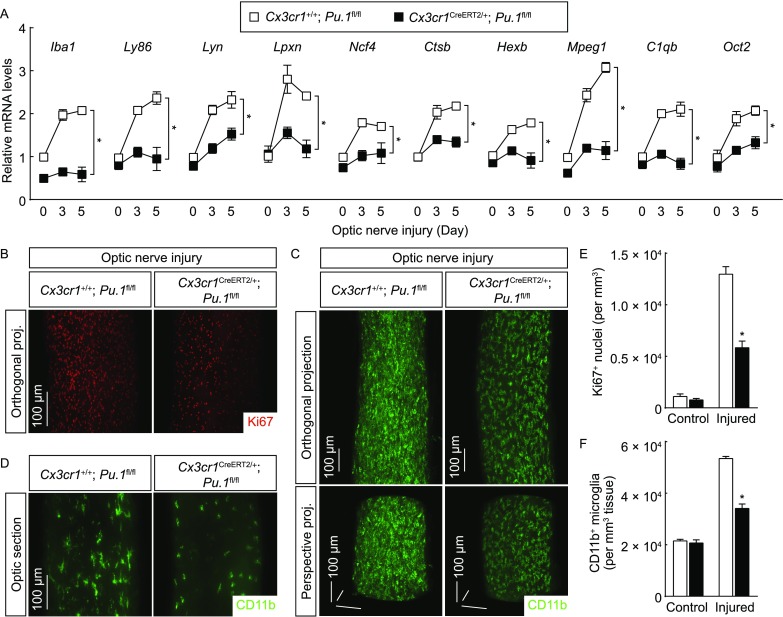
**Post-developmental PU.1 is essential for microglial activation**. *Cx3cr1*^+/+^; *Pu.1*^*fl*/*fl*^ vs. *Cx3cr1*^*CreERT2*/+^; *Pu.1*^*fl*/*fl*^ mice were treated with 4-hydroxytamoxifen to induce the Cre-recombinase activity and then subjected to optic nerve injury. (A) Expression levels of the indicated genes in the optic nerves were examined by qPCR analysis. *n* = 4, mean ± SEM, *P < 0.01 (ANOVA test). (B–F) The control and injured nerves were processed for the whole-tissue immunolabeling of Ki67 (B and E) or CD11b (C, D and F) and imaged on the lightsheet microscope. (B) Representative orthogonal 3D projections of the Ki67-immunolabeled injured nerves. (E) Density of Ki67^+^ nuclei. *n* = 3, mean ± SEM, *P < 0.01 (ANOVA test). (C) Representative orthogonal (upper panels) or perspective (lower panels) 3D projections of the CD11b-immunolabeled injured nerves. (D) Representative optical sections of the CD11b-immunolabeled injured nerves. (F) Density of CD11b^+^ microglia. *n* = 4, mean ± SEM, *P < 0.01 (ANOVA test)

Strikingly, microglial deletion of PU.1 in *Cx3cr1*^*CreERT2-EYFP*/+^; *Pu.1*^*fl*/*fl*^ resulted in the outcomes largely resembling those observed in *Cx3cr1*^*CreERT2-EYFP*/+^; *Irf8*^*fl*/*fl*^ mice, i.e., the up-regulation of microglial activation-related genes was abolished in the injured optic nerves of *Cx3cr1*^*CreERT2-EYFP*/+^; *Pu.1*^*fl*/*fl*^ mice (Fig. 7A), supporting the cooperative action of IRF8 and PU.1 in this microglial response. Also, the microglial proliferation, as well as the increase of microglial population, in response to neurodegeneration was significantly inhibited in *Cx3cr1*^*CreERT2-EYFP*/+^; *Pu.1*^*fl*/*fl*^ mice (Fig. 7B, 7C, 7E and 7F). Moreover, the PU.1-deficient microglia showed the stumpy cell morphology similar to that observed with the IRF8-deficient microglia (Fig. 7D and Movie S4). Taken together, these results have revealed the central transcriptional mechanism of microglial activation under neurodegenerative condition.

## DISCUSSION

In this study, we have exploited the 3D fluorescence imaging to investigate the microglial response to neurodegeneration. Notably, this imaging technique is also applicable to assess other neuropathological processes, e.g., the degeneration of optic axons or the proliferation of oligodendrocytes. Moreover, the 3D fluorescence imaging could be readily extended to examine a variety of glial events in neural tissues beyond the optic nerves, e.g., brain and spinal cord, which would certainly assist in the comprehensive characterization of different CNS diseases.

Previous studies showed that PU.1 is required for the development of myeloid lineage cells, including microglia, in early embryonic stage (Scott et al., 1994; McKercher et al., 1996; Kierdorf et al., 2013). However, whether PU.1 might exert any function in microglial activation in the adulthood had been unexplored. We have found that PU.1 is highly up-regulated in the microglial response to neurodegeneration. Through the microglia-specific deletion of PU.1 in adult mice, we have demonstrated its key post-developmental role in the transition of resting microglia to their activated state. In addition, PU.1 does not act by itself in this process but rather cooperates with IRF8. Again, the genetic approach of microglia-specific deletion of IRF8 in adult mice has substantiated the critical post-developmental role of IRF8 in microglial activation, circumventing the reported developmental deficit of microglia in *Irf8*^−/−^ mice (Kierdorf and Prinz, 2013; Kierdorf et al., 2013).

Importantly, we have elucidated the transcriptional mechanism of IRF8 and PU.1 by profiling their genomic landscapes in activated primary microglia for the first time in the field. We have revealed that IRF8 and PU.1 cross-regulate each other in the process of microglial activation, i.e., PU.1 promotes IRF8 expression by directly targeting the gene locus of *Irf8*, and conversely, IRF8 regulates PU.1 expression through one of the UREs of the *Pu.1* locus. It appears plausible that this positive feedback between IRF8 and PU.1 would effectively sustain their highly enhanced expression, which is essential for the process of microglial activation. Moreover, we have identified that IRF8 and PU.1 dictates the microglial response by cooperatively targeting a large collection of microglial activation-related genes, e.g., the key signaling components of the NF-κB pathway, through the composite IRF-ETS motifs that are enriched at those gene loci. Our work has therefore uncovered the central role of the transcriptional module comprising IRF8, PU.1 and their composite motif in the microglial response to neurodegeneration (Fig. S6).

Given the broad involvement of microglial activation in the CNS diseases, it is a tempting possibility that the transcriptional mechanism we elucidated could also be in action under neuropathological conditions beyond the traumatic injury-induced neurodegeneration. For instance, IRF8 is up-regulated and contributes to the disease progression in the mouse model of experimental autoimmune encephalomyelitis (Yoshida et al., 2014). Also, IRF8 deficiency ameliorates the symptoms in the mouse model of neuropathic pain (Masuda et al., 2012; Inoue and Tsuda, 2018). In light of our current findings, whether PU.1 might be involved, and cooperate with IRF8, in such disease scenarios needs to be determined. Furthermore, future research is warranted to explore the upstream signaling pathway(s) that triggers the up-regulation of IRF8 and PU.1 in response to different neuropathological cues.

In summary, our study has uncovered the central transcriptional mechanism that governs the microglial activation under neurodegenerative condition, which would advance our in-depth understanding of the beneficial and detrimental functions of microglia in the CNS diseases. Also, manipulation of this transcriptional mechanism of IRF8 and PU.1 might become a novel entry point for treatment of divergent neuropathological conditions.

## MATERIALS AND METHODS

### Animal information

All the experimental procedures in mice were performed in compliance with the protocols approved by the Institutional Animal Care and Use Committees (IACUC) of Peking University and Tsinghua University.

The mice utilized in the experiments were 2 to 4 months old. C57BL/6 wildtype mice were purchased from Charles River International. *Sarm1*^−/−^ (JAX 018069, RRID:IMSR_JAX:018069), *Aldh1l1-GFP* (MMRRC 011015-UCD, RRID:MMRRC_011015-UCD), *Cx3cr1*^*CreERT2-EYFP*/*CreERT2-EYFP*^ (JAX 021160, RRID:IMSR_JAX:021160), *Ccr2*^−/−^ (JAX 017586, RRID:IMSR_JAX:017586), *Rosa26-LSL-tdTomato* (JAX 007914, RRID:IMSR_JAX:007914), *Irf8*^*fl*/*fl*^ (JAX 014175, RRID:IMSR_JAX:014175) and *Pu.1*^*fl*/*fl*^ (JAX 006922, RRID:IMSR_JAX:006922) were purchased and bred in-house to generate the littermates for experiments.

Intravitreal injection of the tdTomato-expressing AAV2 was performed as previously described (Yang et al., 2015). The mice were anesthetized, and a customized 33-gauge, 30°-beveled needle attached to a Hamilton syringe was passed through the sclera into the vitreous. For the purpose of sparse labeling of RGCs, 1 × 10^3^ to 5 × 10^3^ transduction units of AAV2 were delivered, which transduced approximately 100 to 500 RGCs in each retina.

Traumatic injury of the optic nerves was performed as previously described (Yang et al., 2015). The mice of indicated conditions were anesthetized, and the topical antibiotic ointment was applied to the eyes. An incision was made on the superior conjunctiva of the left eye, and the optic nerve was exposed by a pair of blunt forceps. The crush injury was performed for 5 s using a pair of fine-tip forceps (Fine Science Tools) at approximately 1 mm distal from the eyeball.

For the parabiosis procedure, each pair of mice was housed together for 1 week before the surgery. The mice were anesthetized, and the skin on one side of each mouse was shaved and prepared with iodine and alcohol. A longitudinal incision was made along the side of each mouse, and the skin was carefully separated from the underlying connective tissues. A longitudinal incision of approximately 10 mm was then made on the exposed peritoneum of each mouse. The incision sites of the two mice were sutured together to establish the connection of vascular systems. In addition, the scapulae on the incision sides of the mice were sutured together to help hold the parabiotic pair. The skin incisions of the mice were closed together by surgical staples. The mice were utilized for experiments 4 weeks after the procedure.

To induce the Cre-recombinase activity in mice of the indicated genotypes, 4-hydroxytamoxifen (Sigma) was formulated in DMSO/Kolliphor-EL/5% sucrose (1:3:6) and administered daily to the mice at 20 mg/kg of body weight via oral gavage for 6 days. To circumvent the issue of microglial death observed around two weeks after the Cre-mediated genetic deletion of PU.1, *Cx3cr1*^*CreERT2*/+^; *Pu.1*^*fl*/*fl*^ mice were subjected to optic nerve injury immediately after 4-hydroxytamoxifen treatment. Otherwise, *Cx3cr1*^*CreERT2*/+^; *Irf8*^*fl*/*fl*^ mice were subjected to optic nerve injury at 4 weeks after 4-hydroxytamoxifen treatment.

### Antibodies

Primary antibodies used in the experiments were rat anti-PECAM1 (BD Biosciences #553370, RRID:AB_394816), rat anti-LYVE1 (Thermo Fisher Scientific #14-0443-82, RRID:AB_1633414), chicken anti-GFP (Aves Labs #GFP-1010, RRID:AB_2307313), rat anti-CD11b (Biolegend #101202, RRID:AB_312785), rabbit anti-RFP (Rockland #600-401-379, RRID:AB_2209751), mouse anti-Iba1 (Millipore #MABN92, RRID:AB_10917271), rabbit anti-Olig2 (Millipore #AB9610, RRID:AB_10141047), goat anti-IRF8 (Santa Cruz #sc-6058, RRID:AB_649510), rabbit anti-PU.1 (Cell Signaling #2258, RRID:AB_10693421), rabbit anti-Ki67 (Millipore #AB9260, RRID:AB_2142366) and mouse anti-p-IκBα (Cell Signaling #9246, RRID:AB_2267145). Alexa Fluor-conjugated secondary antibodies were from Thermo Fisher Scientific.

### 3D fluorescence imaging

The 3D fluorescence imaging of optic nerves was optimized based on the reported iDISCO technique (Renier et al., 2014). The mice were perfused with PBS/50 μg/mL heparin followed by PBS/1% PFA/10% sucrose/50 μg/mL heparin. The optic nerves were dissected out and post-fixed in PBS/0.5% PFA at room temperature for 2 h. The tissues were washed with PBS at room temperature for 30 min and permeabilized with PBS/0.1% TritonX-100/0.1% deoxycholate/10% DMSO/10 mmol/L EDTA (pH 8.0) at room temperature for 6 h. The tissues were blocked with PBS/0.2% TritonX-100/10% DMSO/10 mmol/L EDTA (pH 8.0)/5% normal donkey serum at room temperature overnight. The tissues were immunolabeled with the indicated primary antibodies diluted (1:500) in PBS/0.2% Tween-20/10 μg/mL heparin/5% DMSO/5% normal donkey serum at room temperature for 48 h and washed with PBS/0.2% Tween-20/10 μg/mL heparin at room temperature for 2 h five times. The tissues were then immunolabeled with the corresponding Alexa Fluor-conjugated secondary antibodies diluted (1:1,000) in PBS/0.2% Tween-20/10 μg/mL heparin/5% DMSO/5% normal donkey serum at room temperature for 24 h and washed with PBS/0.2% Tween-20/10 μg/mL heparin at room temperature for 2 h five times.

For the procedure of tissue clearing, the immunolabeled optic nerves were embedded in 0.8% agarose-blocks prepared in PBS. The tissue blocks were dehydrated at room temperature in 20% methanol (diluted in ddH_2_O) for 2 h, 40% methanol for 2 h, 60% methanol for 2 h, 80% methanol for 2 h, and 100% methanol for 1 h twice. The tissue blocks were incubated with the mixture of dichloromethane and methanol (2:1) for 2 h and then with 100% dichloromethane for 30 min twice. The tissue blocks were cleared with 100% dibenzyl-ether for 12 h twice to become ready for the lightsheet fluorescence imaging.

The tissue blocks were imaged on the LaVisionBiotec Ultramicroscope II equipped with the sCMOS camera (Andor Neo) and the 2×/NA0.5 objective (MVPLAPO) covered with the 4-mm-working-distance dipping-cap. Version 144 of the ImSpector Microscope Controller software was supported by LaVisionBiotec. The tissue blocks were immersed in the chamber filled with 100% dibenzyl-ether. For the imaging at 12.6× effective magnification (6.3× zoom), each tissue block was scanned by a single lightsheet (middle position) with a step size of 1 μm. The image stacks were acquired by the continuous lightsheet scanning method without the contrast-blending algorithm.

Imaris (http://www.bitplane.com/imaris/imaris) was used to reconstruct the image stacks obtained from the lightsheet imaging. For display purposes in the figures and movies, a gamma correction of 1.2–1.6 was applied to the raw data (Renier et al., 2014). Movies of the image stacks were generated with the frame rate of 30 fps. Perspective or orthogonal 3D projections of the image stacks were generated as indicated for the representative images shown in figures.

### ChIP-Seq analysis

To determine the transcriptional landscape of IRF8 and PU.1, the procedure of chromatin immunoprecipitation (ChIP) was optimized for the optic nerve tissues. The wildtype mice were subjected to optic nerve injury, and 70 injured optic nerves were acutely harvested. The nerve tissues were immediately washed once in ice-cold PBS and homogenized in ice-cold PBS containing protease inhibitor cocktail (Roche) with a Dounce homogenizer. A final concentration of 1% PFA was added, and the tissue lysates were cross-linked at room temperature for 10 min. The cross-linking reaction was stopped by a final concentration of 125 mmol/L glycine at room temperature for 5 min, followed by centrifugation at 1,000 ×*g* at 4 °C for 5 min to collect the nuclei. The pellet of nuclei was washed with 5 mmol/L PIPES (pH 8.0)/85 mmol/L KCl/0.5% NP-40/protease inhibitor cocktail at 4 °C for 10 min, followed by centrifugation at 2,500 ×*g* at 4 °C for 5 min. The pellet of nuclei was lysed with 50 mmol/L Tris-HCl (pH 8.1)/10 mmol/L EDTA (pH 8.0)/1% SDS/protease inhibitor cocktail at 4 °C for 10 min. The chromatin was then sonicated on the Bioruptor Pico (30 s + 30 s cooling down, 20 cycles) to obtain the genomic DNA fragments ranging from 100 bp to 500 bp, followed by centrifugation at 20,000 ×*g* at 10 °C for 10 min to clear the supernatant.

The sheared chromatin was diluted (1:10) into 16.7 mmol/L Tris-HCl (pH 8.1)/16.7 mmol/L NaCl/1.2 mmol/L EDTA (pH 8.0)/1.1% Triton X-100/0.01% SDS. Then, 10 μL protein A/G magnetic beads (Pierce) equilibrated with 10 mmol/L Tris-HCl (pH 8.0)/1 mmol/L EDTA (pH 8.0)/1 mg/mL BSA was added per 1 mL of the chromatin sample to pre-clear at 4 °C for 1 h. Following the magnetic removal of protein A/G beads, 2 μg of anti-IRF8 or anti-PU.1 antibody was added per 1 mL of the pre-cleared chromatin sample and incubated at 4 °C overnight. Next, 10 μL protein A/G magnetic beads equilibrated with 10 mmol/L Tris-HCl (pH 8.0)/1 mmol/L EDTA (pH 8.0)/1 mg/mL BSA was added per 1 mL of the chromatin sample and incubated at 4 °C for 3 h. The beads were washed at room temperature with 20 mmol/L Tris-HCl (pH 8.1)/150 mmol/L NaCl/2 mmol/L EDTA (pH 8.0)/1% Triton X-100/0.1% SDS for 5 min, 20 mmol/L Tris-HCl (pH 8.1)/500 mmol/L NaCl/2 mmol/L EDTA (pH 8.0)/1% Triton X-100/0.1% SDS for 5 min, 10 mmol/L Tris-HCl (pH 8.1)/250 mmol/L LiCl/1 mmol/L EDTA (pH 8.0)/1% NP-40 for 5 min and 10 mmol/L Tris-HCl (pH 8.0)/1 mmol/L EDTA (pH 8.0) for 5 min twice. The immunoprecipitated chromatin was eluted with 100 mmol/L NaHCO_3_/1% SDS at room temperature for 15 min twice. A final concentration of 200 mmol/L NaCl was added to the pooled eluate, and the cross-linked chromatin was reversed at 65 °C overnight. A final concentration of 50 mmol/L Tris-HCl (pH 6.5)/10 mmol/L EDTA (pH 8.0)/25 μg/mL proteinase K (New England Biolabs) was added to digest the proteins at 37 °C for 1 h. The genomic DNAs were recovered by the QIAquick Purification Kit (Qiagen).

The immunoprecipitated genomic DNAs were sequenced on the Illumina Genome Analyzer II. The accession numbers for the ChIP-Seq data are the Sequence Read Archive (https://www.ncbi.nlm.nih.gov/sra/docs/): SRR6963589 and SRR6963590. The sequencing data were mapped to the mouse genome (mm10) with Bowtie2 (http://bowtie-bio.sourceforge.net/bowtie2/index.shtml/), and the fragments of mapping quality greater than 15 were included for the downstream analysis. IRF8 or PU.1 target sites were called by MACS2 (https://github.com/taoliu/MACS/downloads/). Analysis of the composite motif of IRF8 and PU.1 target sites was performed with MEME (http://meme-suite.org/). Gene Ontology (GO) enrichment analysis of the genes that contained the IRF8-PU.1 co-target sites was performed with PANTHER (http://pantherdb.org/).

### Tissue processing and analyses

For the conventional immunohistochemistry, the mice of indicated conditions were perfused with PBS/50 μg/mL heparin followed by PBS/1% PFA/10% sucrose/50 μg/mL heparin. The optic nerves were dissected out and post-fixed in PBS/0.5% PFA at room temperature for 2 h. The tissues were cryopreserved in PBS/30% sucrose at 4 °C overnight, then processed for 5-μm cryosectioning. The sections were immunostained with the indicated primary antibodies and corresponding Alexa Fluor-conjugated secondary antibodies, and then imaged by the fluorescence microscopy.

For the qPCR analysis of gene expression, the optic nerves were acutely dissected from the mice of indicated conditions. Total RNAs of the nerve tissues were extracted by the RNeasy Mini Kit (Qiagen), and then reverse-transcribed and analyzed by the SYBR Green Real-Time PCR Kit (Thermo Fisher Scientific).

### Electrophoretic mobility shift assay

Mouse IRF8 and PU.1 cDNAs were cloned into the pcDNA3 vector with the Flag-tag. The plasmids were transfected into HEK293T cells, and recombinant IRF8 and PU.1 proteins were purified from the nuclear extracts of transfected cells with anti-Flag M2 Affinity Gel (Sigma). For each DNA probe, the sequence was derived from the IRF8-PU.1 co-target site at the indicated gene locus. The complementary pair of 5′-biotin-labeled primers were synthesized and annealed for each probe. The electrophoretic mobility shift assay (EMSA) was performed with the LightShift Chemiluminescent EMSA Kit (Thermo Fisher Scientific). For the supershift assay, anti-PU.1, anti-IRF8 or control IgG was added after the formation of the composite-motif DNA/IRF8/PU.1 complex, and the biochemical reactions were incubated at 4 °C for 20 min.

### Statistical methods

To quantify the traumatic injury-induced neurodegeneration, the tdTomato-labeled axons were manually traced in the 3D-reconstructed image of each nerve tissue, with any sign of axonal fragmentation scored as degeneration. To quantify the density of Olig2^+^ oligodendrocyte nuclei, three 100 μm × 100 μm × 100 μm volumes were randomly selected along the 3D-reconstructed image of each nerve tissue, and the immunolabeled nuclei in each cubic volume were manually counted. To quantify the density of Ki67^+^, IRF8^+^ or PU.1^+^ nuclei, four 100 μm × 100 μm × 100 μm volumes were randomly selected along the 3D-reconstructed image of each nerve tissue, and the immunolabeled nuclei in each cubic volume were counted. To quantify the density of Iba1^+^ or CD11b^+^ microglia, four 100 μm × 100 μm × 100 μm volumes were randomly selected along the 3D-reconstructed image of each nerve tissue, and the immunolabeled microglia in each cubic volume were counted.

Student’s *t*-test or ANOVA test was performed using GraphPad Prism (http://www.graphpad.com/scientific-software/prism). Statistical details of the experiments can be found in the figure legends.

## Electronic supplementary material

Below is the link to the electronic supplementary material.

Supplementary material 1 (MOV 3185 kb)

Supplementary material 2 (MOV 5698 kb)

Supplementary material 3 (MOV 4827 kb)

Supplementary material 4 (MOV 5109 kb)

Supplementary material 5 (XLS 58 kb)
